# A Unified Proteochemometric Model for Prediction of Inhibition of Cytochrome P450 Isoforms

**DOI:** 10.1371/journal.pone.0066566

**Published:** 2013-06-17

**Authors:** Maris Lapins, Apilak Worachartcheewan, Ola Spjuth, Valentin Georgiev, Virapong Prachayasittikul, Chanin Nantasenamat, Jarl E. S. Wikberg

**Affiliations:** 1 Department of Pharmaceutical Biosciences, Uppsala University, Uppsala, Sweden; 2 Center of Data Mining and Biomedical Informatics, Faculty of Medical Technology, Mahidol University, Bangkok, Thailand; 3 Department of Clinical Microbiology and Applied Technology, Faculty of Medical Technology, Mahidol University, Bangkok, Thailand; Concordia University Wisconsin, United States of America

## Abstract

A unified proteochemometric (PCM) model for the prediction of the ability of drug-like chemicals to inhibit five major drug metabolizing CYP isoforms (*i.e.* CYP1A2, CYP2C9, CYP2C19, CYP2D6 and CYP3A4) was created and made publicly available under the Bioclipse Decision Support open source system at www.cyp450model.org. In regards to the proteochemometric modeling we represented the chemical compounds by molecular signature descriptors and the CYP-isoforms by alignment-independent description of composition and transition of amino acid properties of their protein primary sequences. The entire training dataset contained 63 391 interactions and the best PCM model was obtained using signature descriptors of height 1, 2 and 3 and inducing the model with a support vector machine. The model showed excellent predictive ability with internal AUC = 0.923 and an external AUC = 0.940, as evaluated on a large external dataset. The advantage of PCM models is their extensibility making it possible to extend our model for new CYP isoforms and polymorphic CYP forms. A key benefit of PCM is that all proteins are confined in one single model, which makes it generally more stable and predictive as compared with single target models. The inclusion of the model in Bioclipse Decision Support makes it possible to make virtual instantaneous predictions (∼100 ms per prediction) while interactively drawing or modifying chemical structures in the Bioclipse chemical structure editor.

## Introduction

There are close to sixty Cytochrome P450 enzymes (CYPs) present in humans, where they facilitate oxidative metabolism of endogenous substances and xenobiotics. Two-thirds of currently used drugs are cleared by metabolism, and seven CYPs contribute to the clearance of more than 90% of these compounds. The major drug-metabolizing isoforms are CYP1A2 (estimated to catalyze metabolism for 2% of drugs), CYP2B6 (4%), CYP2C9 (10%), CYP2C19 (5%), CYP2D6 (28%), CYP2E1 (4%), and CYP3A4 (47%) [1], [2]. Being broadly specific with respect to their substrates, CYPs are also susceptible to inhibition by a large variety of chemical compounds. The results of a recent large-scale screening against five CYP isoforms identified that the majority of compounds in a typical chemical library cross-inhibited several isoforms, while only 7% of the compounds did not inhibit any of the isoforms [3].

CYP inhibition leads to decreased elimination and/or changed metabolic pathways of their substrates, which is the major cause of adverse drug-drug interactions [2], [4]. It is therefore essential to identify potential problems with CYP liability at an early stage in drug discovery. During the last decade, techniques for high throughput *in vitro* screening of CYP inhibition were developed and implemented on a broad scale in the drug discovery pipelines of pharmaceutical companies, as well as much open data has accumulated through academic research initiatives (e.g. PubChem Bioassays AID 410 and 1851) [5]. The collected data has enabled development of structure-activity relationship models for *in silico* prediction of CYP inhibition. Thus, Vasanthanathan et al. [6] and Novotarskyi et al. [7] recently developed large-scale single target models for CYP1A2 isoform, and Cheng and co-workers [8] created single target models for five CYP isoforms (*i.e.* QSAR models). These models show good predictive performances, but have the disadvantage that they are not implemented as publicly available services. Another deficiency of these models (except the work by Cheng et al. [8]) is the use of molecular descriptors that are calculated by commercial software packages, which does not allow implementation of the models in free, open source software.

All previous studies created structure-activity models for one CYP subtype at a time. This may be a suboptimal approach since the inhibition profiles of CYPs largely overlap. A more general technique is proteochemometrics (PCM), a modeling technology that we introduced some time ago [9] to study similarities and differences in molecular interaction mechanisms of groups of related proteins [10], [11]. PCM creates unified models for multiple proteins interacting with multiple ligands by correlating the interaction data to descriptors of both sets of interacting entities. Previous studies on G-protein coupled receptors, proteases, protein kinases, and other protein classes have shown PCM to be able to predict activity profiles of untested chemical compounds as well as activity profiles of untested proteins [10]–[14]. In this study, we aimed to create a unified PCM model for CYPs suited for drug profiling using free, open-access software and make the model publicly available for predictions using earlier developed open source Bioclipse Decision Support system [15].

## Materials and Methods

### Datasets

#### Dataset for model development

**Figure 1:**
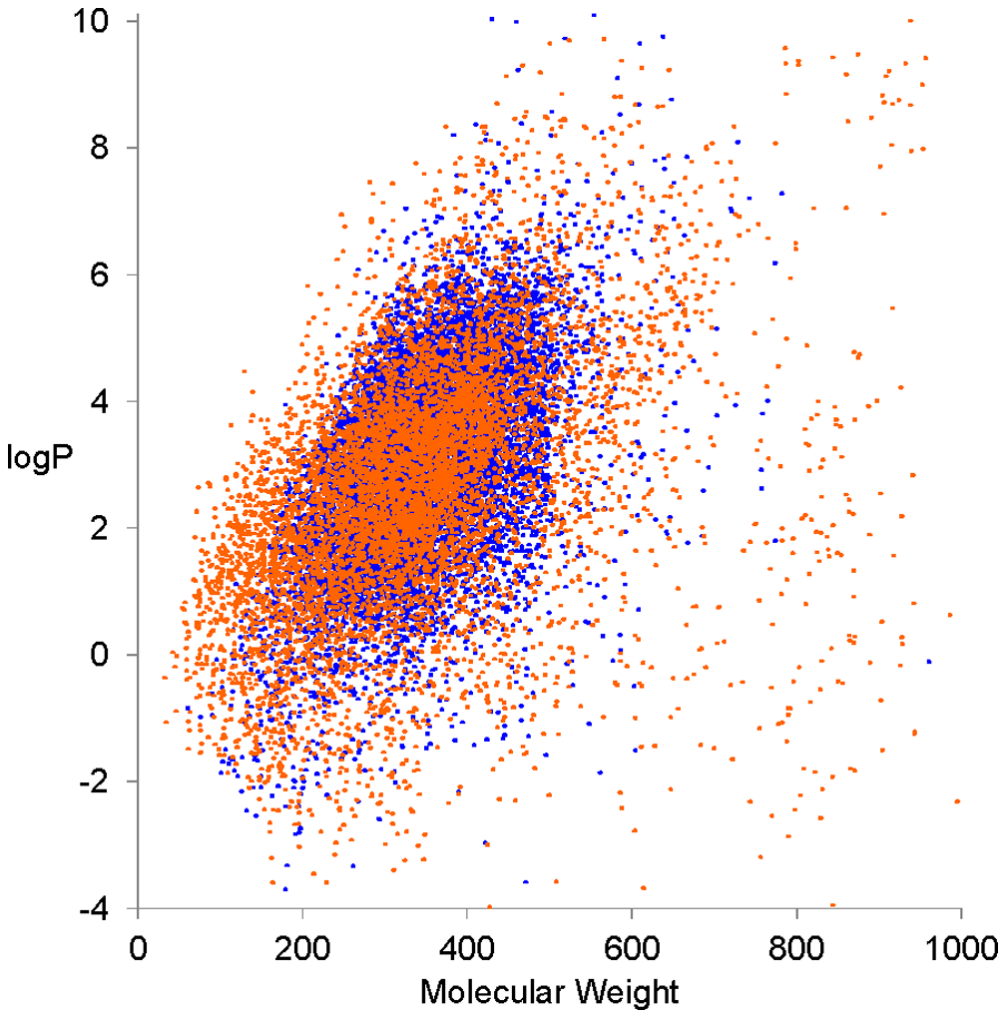
Chemical space covered by the dataset . The graph shows logP versus the molecular weight of the compounds. Work set compounds are shown as blue dots and prediction set compounds as red dots. The logP was calculated by Dragon 5.5 software (Talete.srl).

We used PubChem BioAssay dataset AID = 1851 containing data for inhibition of five major CYP isoforms (CYP1A2, CYP2C9, CYP2C19, CYP2D6 and CYP3A4) by 17 143 chemical compounds [3], [5]. Inorganic compounds, non-covalent inhibitors and compound mixtures were removed from the dataset, leaving 16 359 compounds. The dataset classified compounds as active or inactive for the respective CYP, and the activity cutoff was set to AC_50_ = 10 µM (AC_50_, “activity concentration 50”, refers to the concentration that is required to elicit half-maximal effect). However, in cases when the dose-response curve for a compound showed poor fit or the inhibition efficacy was below 60%, the assay results were regarded as inconclusive. Thus, not all compounds had activity outcomes for all five CYP isoforms, but the dataset contained all-in-all 63 391 compound-CYP combinations. The fraction of compounds found to be active ranged from 19% for CYP2D6 to 46% for CYP1A2 (Table 1). The dataset comprised drugs and drug-like compounds. The chemical space revealed that the majority of compounds had molecular weight below 500 daltons and logP below 5 (Figure 1).

**Table 1:** Composition of the dataset.

CYP isoform	Tested compounds	Activecompounds	Active only against this CYP	Active also against:				
				1A2	2C9	2C19	2D6	3A4
1A2	12 634	5 838 (46%)	1 395		2 011	3 252	1 272	2 565
2C9	12 264	4 024 (33%)	370	2 011		3 022	800	2 115
2C19	12 834	5 763 (45%)	562	3 252	3 022		1 363	3 012
2D6	13 276	2 545 (19%)	622	1 272	800	1 363		1 252
3A4	12 383	5 165 (42%)	889	2 565	2 115	3 012	1 252	

#### Dataset for model validation

Dataset for external validation of the model was obtained from Cheng et al. [8] and comprised 8 988 compounds tested on at least one of the five CYP isoforms studied herein. In this dataset, compounds are characterized by the so-called PubChem activity score,and are regarded as inhibitors if the activity score ranges between 40 and 100. PubChem activity score is assigned based on an AC_50_ value, which is combined with a measure for completeness of a dose-response curve and efficacy of inhibition, where a larger value indicates higher inhibitory activity and/or higher confidence in inhibitory assay result. Compounds with activity score equal to 0 are considered as non-inhibitors while compounds with activity score above 0 and up to 40 are considered as inconclusive and therefore removed from the dataset. Under these constraints, the fraction of inhibitors obtained in the dataset ranges from 19% for CYP2D6 to 62% for CYP1A2 [8]. The dataset for model validation is hereinafter termed ‘external test set.’

### Description of Chemical Compounds

All compounds were obtained as SMILES strings and converted to SDF format by open source Bioclipse workbench version 2.6 software [16]–[18]. The compounds were thereafter encoded by molecular signatures [19], which were generated by Bioclipse 2.6.

An *atomic signature* is a canonical representation of the atom’s environment up to a predefined height (*i.e.* the bond number to the neighboring and next-to neighboring atoms that the signature spans). Only heavy atoms but not hydrogens are considered in calculation of signatures. Signatures distinguish between single, double, and triple bonds, as well as between aromatic and aliphatic atoms in the atom’s environment. (There is no further distinction of atoms depending on chirality or hybridization state, however). Presence of the same atomic signatures in several compounds indicates thus that these compounds share identical or similar 2D structural fragments or features.

A *molecular signature* constitutes a vector of occurrences of all atomic signatures in the dataset. In the present dataset, we found 460 atomic signatures of height one, 13 386 atomic signatures of height two, and 67 168 of height three. In other words, molecular signatures of heights one, two and three comprised in our dataset vectors of 460, 13 386, and 67 168 integers.

### Description of CYP Isoforms

We encoded the five CYP enzymes by alignment-independent description of composition and transition of amino acid properties in the protein primary sequences as proposed by Dubchak and coworkers [20]. The descriptors are based on seven amino acid properties: 1) hydrophobicity, 2) normalized van der Waals volume, 3) polarity, 4) polarizability, 5) charge, 6) secondary structure, and 7) solvent accessibility. For each of these seven attributes, amino acids are assigned to three classes. As there are seven attributes and three classes, 7×3 = 21 composition descriptions can be computed, representing percentages of the attributes/classes in the protein sequence. The transition descriptors represent frequencies with which an attribute changes class along the sequence, *e.g.*, a class 1 amino acid is followed by a class 2 amino acid or *vice versa*. As there are three possible transitions between classes, 7×3 = 21 transition descriptors can be calculated. Composition and transition descriptors were computed by using PROFEAT (Protein Feature) web server [21].

### Methods of Correlation of Compound and CYP Descriptors to the Activity Data

#### Support Vector Machine (SVM)

SVM is a machine-learning technique for classification or regression that uses linear or non-linear kernel-functions to project the data into a high-dimensional feature space. Correlation is performed in this hyperspace based on the structural risk minimization principle; *i.e*., aiming to increase the generalization ability of a model [22]. We induced PCM models by applying the commonly used Gaussian radial basis function kernel. SVM requires fine-tuning of several parameters to obtain an optimal model, the most important being the width of the kernel function γ and the error penalty parameter C. We found optimal γ and C by performing grid search and evaluating resulting models by five-fold cross-validation. SVM models were created by libSVM software as accessed from Weka 3.7 [23]. The final model was implemented in Bioclipse Decision Support system using e1071 package from the R software environment accessed under Bioclipse 2.6.

#### K-Nearest Neighbor method (k-NN)

The k-NN algorithm predicts the class of a test set object based on the class membership of its k most similar training set objects. We induced k-NN models using Weka 3.7 software. To obtain optimal k-NN models one must find the optimal number of neighbors considered to predict class membership. Moreover, in PCM modeling the results are influenced by the scaling of compound descriptors, relative to the scaling of protein descriptors. To obtain optimal models, we systematically varied scaling of the two descriptor blocks and the number of considered neighbors while estimation of the prediction accuracy for the resulting models was performed by means of five-fold cross-validation.

#### Random Forest

Random Forest (RF) is a classifier that consists of multiple decision trees. A decision tree is made of nodes and branches. At each node the dataset is split based on the value of some attribute that is selected so that the instances of different classes are predominantly moved to different branches. Classification starts at the root node and is performed by passing the instances along the tree to leaf nodes. To introduce diversity between the trees of a random forest, a small subset of all attributes is randomly selected to take decisions at each node of each tree. The classification is performed by considering results of all trees by a majority vote. We generated RF using the RandomForest classifier of Weka 3.7. The optimal size of the forest and the number of attributes to consider at each node were found by performing five-fold cross-validation.

### Assessment of the Quality of the Models

We assessed the predictive ability of the models by performing cross-validation and external predictions. We used two statistical measures: the overall prediction accuracy and the area under the Receiver Operating Characteristic (ROC) curve.

Accuracy is simply the percentage of correctly classified instances and is calculated as
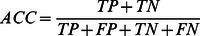
where TP is the number of true positives, TN is the number of true negatives, FP is the number of false positives or over-predictions, and FN is the number of false negatives or missed predictions.

However, accuracy is not an optimal measure of model performance if the dataset is unbalanced (*i.e.* sizes of the classes are unequal) or if certain errors are to be considered more serious than others (*e.g.* false negatives compared to false positives). In contrast to accuracy, the area under the Receiver Operating Characteristic curve is a measure of the discriminatory power that is insensitive to changes in class distribution and the costs of making certain errors. A ROC curve is obtained by calculating *sensitivity* and *specificity* at various discrimination threshold levels. Sensitivity is the fraction of true positives among all positively classified instances (the true positive rate) and is calculated as:
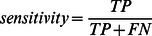



Specificity is the true negative rate and is calculated as:
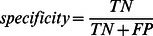



An increased sensitivity is always accompanied by decreased specificity. A ROC curve is plotted as sensitivity versus 1-specificity, at varied discrimination cut-offs. An area under the ROC curve (AUC) close to 1 means that the classifier can perfectly separate the two classes, whereas an area 0.5 indicates that the classifier does not perform better than random guessing.

## Results and Discussion

### Activity Data

The success of PCM modeling depends to a large extent on partial overlaps of the activity profiles of the studied proteins, or its multi-covariance, when the data are quantitative. An overview of the activity data is given in Table 1. As seen, more than half of the compounds that are inhibitory on CYP1A2 inhibit also CYP2C19 (3 252 of 5 838). CYP1A2 also shares more than 40% of its inhibitors with CYP3A4 and 35% with CYP2C9. Inhibitors of CYP2C9 also tend to be active against CYP2C19 (3 022 of 4 024, i.e. 75%), although the opposite tendency is less pronounced, the total number of inhibitors of CYP2C19 being 5 763 (*i.e,* only 52% of them, that is 3 022 of 5 763, inhibit also CYP2C9). CYP2C19 also shares 3 012 inhibitors with CYP3A4, but only 1 363 inhibitors with CYP2D6. In fact, CYP2D6 shows the most distinct inhibition profile, having fewer inhibitors than the other isoforms, and about ¼ of them being specific for only this isoform.

In analyzing the activity data one should take into account that not all compound–CYP combinations had conclusive assay results; hence, the fraction of compounds inhibiting several CYPs is expected to be even greater than estimated in the table. Thus, taken together, the interaction data suggests that the dataset is well suited for simultaneous PCM modeling of compound interactions with all five CYP isoforms.

### Proteochemometric Modeling

We developed several PCM models for the inhibition of five isoforms of CYP450 enzyme using the dataset of 16 359 organic compounds, comprising totally 63 391 organic compound–CYP isoform combinations. CYP isoform–inhibitor combinations were assigned the category membership +1, and CYP isoform–non-inhibitor combinations were assigned the category membership −1. The structures of chemical compounds were encoded by molecular signature descriptors and the CYPs were represented by descriptors of amino acid property composition and transition in their primary sequences. Description of compound-CYP combinations was formed by concatenation of the respective compound and CYP descriptions. PCM models correlating the thus obtained compound-CYP description to the activity data were developed using three non-linear data analysis techniques: Support Vector Machine, Random Forest, and k-Nearest Neighbor method. These three techniques not only perform binary classification (i.e. −1/+1) but also provide class probability estimates, i.e. quantitative predictions in the range between −1 and 1. To estimate the predictive ability of the models we calculated accuracy (percentage of correctly classified instances) at zero threshold level as well as area under the ROC curve (AUC), which is obtained by plotting model sensitivity versus specificity at varying discrimination thresholds. The models were validated internally by performing five-fold cross-validation as well as by use of the external test set, as described under Materials and Methods.

We created PCM models based on the compound description of increasing complexity (i.e. increasing height of molecular signatures) (Table 2). The Random Forest technique, which employ molecular signatures of height 1, produced an excellent model with internal AUC = 0.900 and external AUC = 0.933. The model improved further by adding higher signatures, which gave internal AUC = 0.918 and external AUC = 0.946. The best Support Vector Machine model was obtained by combining signatures of height 1, 2, and 3, which gave the internal AUC = 0.923 and external AUC = 0.940; its performance thus being about the same as the best random forest model. The performance of k-Nearest Neighbor approach was inferior to the two other methods; all models with height 1, 2, and 3 showing similar performances with AUCs ranging 0.860–0.870.

**Table 2:** Summary of the performance of PCM models.

Classification method	Maximum height of signature	Cross-validation		External prediction	
		accuracy (%)	AUC	accuracy	AUC
Support Vector Machine	1	83.73	0.905	82.52	0.883
	2	85.13	0.920	87.04	0.922
	3*	85.68	0.923	88.33	0.940
Random Forest	1	83.03	0.900	87.53	0.933
	2	84.35	0.915	88.32	0.941
	3	84.16	0.918	88.63	0.946
k-Nearest Neighbors	1	78.97	0.865	80.38	0.866
	2	79.56	0.870	80.10	0.868
	3	78.48	0.860	79.54	0.865

* Model implemented in Bioclipse Decision Support at www.cyp450model.org.

**Figure 2:**
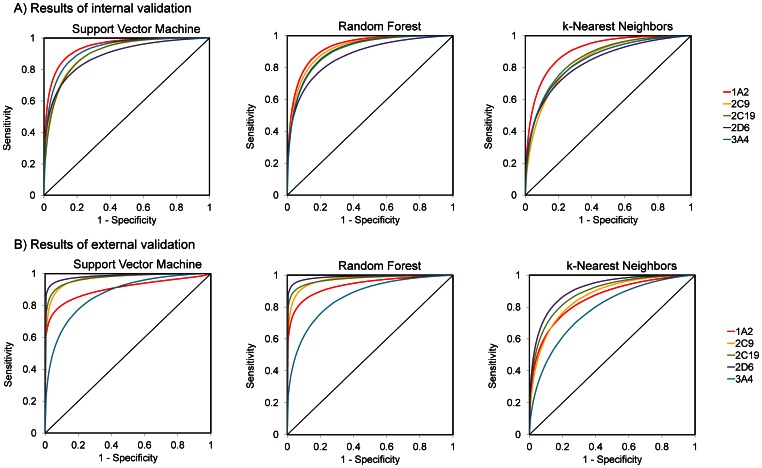
ROC curves for proteochemometric models of CYP inhibition. Shown are results from models induced by Support Vector Machine, Random Forest, and k-Nearest Neighbor algorithms. Chemical compounds were described by molecular signatures of height 1–3 in all three models. Panel A presents ROC curves obtained during five-fold cross validation and panel B presents ROC curves obtained from the predictions for the external dataset. The area under the ROC curve (AUC) is a measure of the discriminatory power of a model. The numerical values of AUC of each model are given in Table 2.

ROC curves for individual CYP isoforms, as obtained from cross-validation of the models, are presented in Figure 2 Panel A. In the SVM model, at 80% sensitivity level the specificities for the five isoforms ranged 80 to 92%, while in the RF model, the specificity is slightly lower for CYP2D6. The figure confirms that the discriminative ability of k-NN model is slightly lower, except for the CYP1A2 isoform.

The results from the external validation are even more impressive. For three of the isoforms the specificity at 80% sensitivity level is 97–99% for both the SVM and RF models (Figure 2 Panel B). The superior results compared with that of the internal validation may be explained in part by the way the external test set was created. As described under Materials and Methods, compounds of the external test set were classified as active/inactive based on their PubChem activity scores, which is a classification that may not be directly comparable to AC_50_ values. Further on, scores between 0–40% were considered as inconclusive and compounds falling within this range were removed from the dataset, a situation which would likely make the predictions easier.

However, the external predictions for CYP1A2 are not superior to the cross-validation results. The shape of the ROC curve indicates that for this isoform very high sensitivity can be achieved only at a cost of low specificity. Remarkably, CYP1A2 is the only one of the five enzymes for which the fraction of inhibitors in the external test set is higher than in the work set (64% versus 46%).

For one of the five isoforms, CYP3A4, the area under the ROC curve as obtained from external predictions is lower than AUC from internal validation (0.865 versus 0.907 in RF model and 0.889 versus 0.927 in SVM model). This can be attributed to differences between the used activity criteria or to differences in the compositions of the two datasets. In particular, the fraction of inhibitors of CYP3A4 in the test set was below 30% as compared to 42% in the dataset as used for model development. It is also known that inhibitor effects may be influenced by the assay conditions; e.g. differences may arise from the use of different substrates [24].

In an earlier study by Cheng et. al. [8], the same dataset was exploited for external validation for single target models, and the models also performed best for CYP2C9, CYP2C19, and CYP2D6 where the AUC values being in the range 0.842–0.886. The AUC for CYP3A4 was, however, only 0.79, indicating that this enzyme is indeed most promiscuous and therefore difficult to predict (in fact, CYP3A4 is involved in metabolism of almost half of all currently used drugs). It is notable that our proteochemometric model seems to outperform the earlier single target models in terms of predictive accuracy.

### Implementation of the Proteochemometric CYP Model in Bioclipse Decision Support

Bioclipse Decision Support provides a workbench where scientists can download and execute predictive models on chemical structures [15]. We packaged the proteochemometric model developed with the Support Vector Machine using molecular signatures of height 1–3 (model marked by an asterisk in Table 2) and made it available for predictions from within Bioclipse Decision Support. Bioclipse [16]–[18] and Bioclipse Decision Support [15] are available as open source under the Eclipse Public License (EPL). Binary downloads of Bioclipse for Windows, Linux, and Mac OS X are available from http://www.bioclipse.net and source code is available from https://github.com/bioclipse. After downloading and unpacking Bioclipse, users can install the proteochemometric CYP450 model as is described in full detail in on the website www.cyp450model.org [25], accompanying this study.

**Figure 3:**
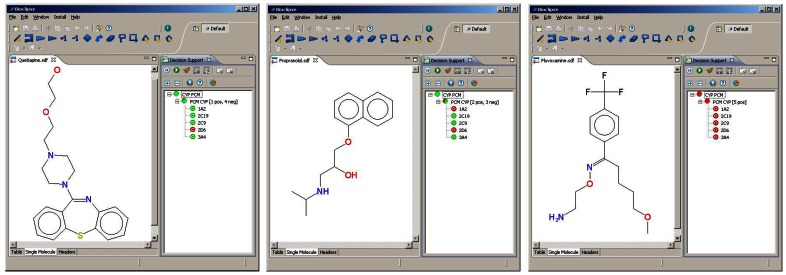
Screenshots of Bioclipse Decision Support showing the predictions of proteochemometric model. Left panel) Model predicts that Quetiapine inhibits CYP2D6. Middle panel) Model predicts that Propranolol inhibits CYP1A2 and CYP2D6. Right panel) Model predicts that Fluvoxamine inhibits all five CYPs.

Users are then able to draw or import chemical structures and predict CYP inhibition (see Figure 3, for examples). The implementation is fast, taking only about 100 ms for an average chemical structure.

### Predictions of Inhibition of CYP Isoforms that are not Represented in PCM Models

We also created proteochemometric models for five reduced datasets, leaving out one CYP isoform at a time and then used the reduced models to predict the activities of the compounds against the excluded isoform. This was done to test the hypothesis that PCM models can be used to predict inhibition of CYP isoforms that have not been subjected to extensive experimental testing and thus lack a bulk of experimental data.

The models were validated using two test sets: Test Set A composed of compound–CYP combinations where CYP isoform was excluded from the dataset but the compound could still be present in it *(i.e.* data from BioAssay AID = 1851); Test Set B included compound–CYP combinations where neither the CYP isoform nor compound were present in the dataset used for model building (*i.e.* data from dataset collected by Cheng et al. [8]).

**Figure 4:**
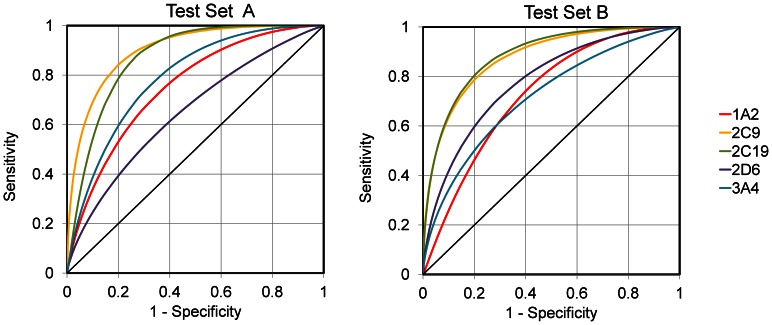
ROC curves for proteochemometric models built using data for 4 of 5 CYP isoforms and performing predictions for the CYP isoform not present in the model. The models were induced by the Random Forest algorithm using molecular signatures of height 1–3. Compounds of Test Set A are from dataset of BioAssay AID = 1851; most of them are tested on several CYP isoforms and thus remained in the dataset and used during model building. Test Set B contains compounds from external dataset.

The best models for the five reduced datasets were obtained by Random Forest, the average AUC for Test Sets A being AUC = 0.792 and for Test Sets B AUC = 0.797. ROC areas for each of the CYP isotypes are shown in Figure 4. The best predictions are for CYP2C9 and CYP2C19, which in fact are phylogenetically closest of the studied CYP isoforms. As seen in the figure, for these CYPs at 80% sensitivity level, specificity also exceeds 80%. For CYP1A2 and CYP3A4, the predictions are moderately good where at 80% sensitivity the specificity being about 60% for Test Set A and 50% for Test Set B. The lowest prediction accuracy (AUC = 0.66) in Test Set 1 is for the CYP2D6. This can be explained by the markedly distinct inhibition profile of this isoform as compared to the others. In particular, of the compounds inhibiting all other CYP isoforms they are tested on, only 35% also inhibit CYP2D6. The presence of these compounds in both the work set and the test set apparently leads to mispredictions. This is evident from comparison with the excellent prediction results for the Test Set B (AUC = 0.79), comprising novel unseen compounds not biasing the predictions towards false-positive results.

## General Discussion and Conclusions

We have shown in several earlier studies the advantage of PCM over single target modeling approaches in predictive modeling [9], [10], [26], [27]. PCM models can host several hundred targets in one unified model thereby aiding the development of broad predictive models [10]. Furthermore, the PCM approach results in more stable and predictive models for datasets comprising of only a few targets when compared to single target models. A value-added benefit is also that PCM models make richer interpretations possible [9]. The performance improvement afforded by PCM models can be attributed to the richer information content when data for multiple targets and compound series are included into a unified model. We have even shown earlier that PCM is useful for the modeling of inhibition of multiple CYP-isoforms and gives higher predictive ability than conventional single target approaches [27]. However, the latter model was built on a limited set of only 375 compounds, which made it not practically useful for drug profiling.

In this study, we aimed to develop a proteochemometric model for the prediction of the inhibition of five major drug-metabolizing CYP isoforms that are suited for general drug profiling. All stages of the modeling are performed using open source software, and the best-performing model is hosted in the Bioclipse Decision Support. In this model, chemical compounds are represented by molecular signatures of height 1–3 comprising more than 80 000 atomic signatures. Thus, the predictions are based on the presence and count of multiple overlapping molecular fragments of various sizes.

On an average, the dataset compounds contained 13.6 atomic signatures of height one (i.e. non-zero values of the molecular signature). The corresponding average values for height two are 19.3 and for height three 21.2 atomic signatures per compound (these numbers for individual compounds are roughly proportional to the number of atoms in the molecule). Thus, despite the large sizes of molecular signatures of heights two and tree, the size of the whole dataset in sparse representation does not become as extensive as to make the data storage and computations too resource demanding to not be affordable in reasonable time at reasonable cost.

We built PCM models using three non-linear algorithms for the correlation of descriptors of chemical compounds and CYP isoforms to the compound–CYP inhibition data. Non-linear methods allow PCM to account for complementarities of ligand and protein properties that are required for their interactions (for *in depth* discussion of technical aspects of PCM we refer to [10], [11]). Of the three methods, Support Vector Machines and Random Forest were comparable and yielded models with very good predictive performances; the accuracies ranging between 84–88%, and AUC being above 0.9 for both cross-validation and external predictions.

The main advantage of hosting the model on Bioclipse Decision Support is that users can draw or import chemical structures in various file formats, modify the structures and perform predictions in an interactive mode where the average time for recalculations being about 100 ms for a medium-sized molecule. This is in vast contrast to current workflows for typical QSAR and proteochemometric studies where chemical structure management, descriptor calculation, and statistical analysis are treated as separate steps and performed by non-integrated software packages, which leads to low throughput and even lack of possibility of performing predictions for new compounds and updating the models when new data become available. By contrast, the Bioclipse platform makes all of this easy in an interactive way. Bioclipse is a fully scriptable work-bench, which provides means to plug-in various open source cheminformatics, bioinformatics, and data analysis tools [10], [15]–[18]. The Bioclipse platform facilitates streamlined modeling without any manual, time consuming, and error prone steps of data conversion and transfer between various software packages joined in *ad hoc* fashions. An important feature of Bioclipse Decision Support [15] is also the ability to select and run multiple predictive models at the same time, making it easily available to complement the CYP model with models for other endpoints such as activity on targets and off-targets, toxicity, and ADME (see [24]).

At present a bulk of inhibition data has been collected for just five of about sixty CYP isoforms present in humans. Interestingly, CYP isoforms that metabolize only few drugs can be inhibited by a large array of compounds. For example, 46% of compounds from our analyzed dataset inhibited CYP1A2, 45% inhibited CYP2C19, and 42% inhibited CYP3A4, although the former two isoforms mediate the metabolism of much fewer drugs than the latter one (2%, 5%, and 48%, respectively). A tendency that the fraction of inhibitors is higher than the fraction of substrates allows for the possibility that other CYP isoforms are also inhibited by a substantial fraction of presently used drugs. Aside from the five CYPs studied herein, several other isoforms have gained increased attention as potentially important drug-metabolizing enzymes [4], [28], [29] (to mention a few examples of metabolic pathways of widely-used drugs, the metabolism of nicotine is predominantly mediated by CYP2A6, metabolism of ethanol by CYP2E1, metabolism of antimalarial drug amodiaquine and several oral antidiabetics by CYP2C8, and metabolism of antidepressant bupropion and HIV reverse transcriptase inhibitor efavirenz by CYP2B6 [4]). Predictions of PCM models could remedy the absence of experimental data for drug interactions with these and other CYP isoforms. Although our dataset is comprised of only five enzymes, models leaving-out one CYP at a time showed the potential of PCM for such cross-CYP isoform predictions. Updating the PCM model with more isoforms as more data becomes available, thus affords the great advantage that over time a single unified PCM model providing coverage for the entire span of all CYP isoforms could be achieved.

Another important point is that the presently available drug-CYP inhibition data do not account for high genetic polymorphism of these enzymes, which gives rise to extensive inter-individual variation in human drug metabolism [30]. Such variation poses a problem for the drug industry owing to the adverse effects, therapeutic failure, and toxicity in those receiving treatment. However, we foresee that data for mutated CYP variants will be collected in the near future and that generalized PCM models could have a role to analyze drug interaction data with multiple genetically diverse CYP populations, thus enabling *a priori* predictions of individuals and populations based on their genetic make-up that might respond adversely or even with idiosyncratic drug reactions to drug combinations and a drug in development.
